# Rapid growth of esophageal papilloma after chemoradiotherapy for esophageal cancer

**DOI:** 10.1016/j.igie.2025.09.012

**Published:** 2025-09-24

**Authors:** Kentaro Kawamura, Yutaka Okagawa, Norito Suzuki, Masahiro Hirakawa, Kohichi Takada

**Affiliations:** Department of Oncology, Sapporo Medical University Hospital, Sapporo, Japan

A 62-year-old man with a history of radiotherapy for hypopharyngeal cancer was diagnosed with advanced esophageal squamous cell carcinoma. Findings of an esophagogastroduodenoscopy revealed an ulcerative-type tumor located in the cervical esophagus and a 10-mm white, elevated lesion distal to the tumor, endoscopically diagnosed as an esophageal papilloma (Fig. 1A). Because of a high surgical risk, the patient underwent chemoradiotherapy. At 5-month follow-up, the primary tumor had completely regressed; however, the white elevated lesion had rapidly enlarged and exhibited a villous morphology (Fig. 1B). Magnified endoscopy with narrow-band imaging revealed increased caliber and the density of intrapapillary capillary loops (Fig. 1C). Although malignant features were absent, endoscopic submucosal dissection was performed for diagnostic treatment because of the rapid growth of the lesion. Hematoxylin and eosin staining revealed papillary proliferation of stratified squamous epithelium with dilated stromal vessels and inflammatory infiltration, without cytological atypia (Fig. 1D). The final diagnosis was esophageal papilloma. Although the underlying mechanism remains unclear, this case suggests that esophageal papilloma may rapidly increase after chemoradiotherapy.

**Figure 1:**
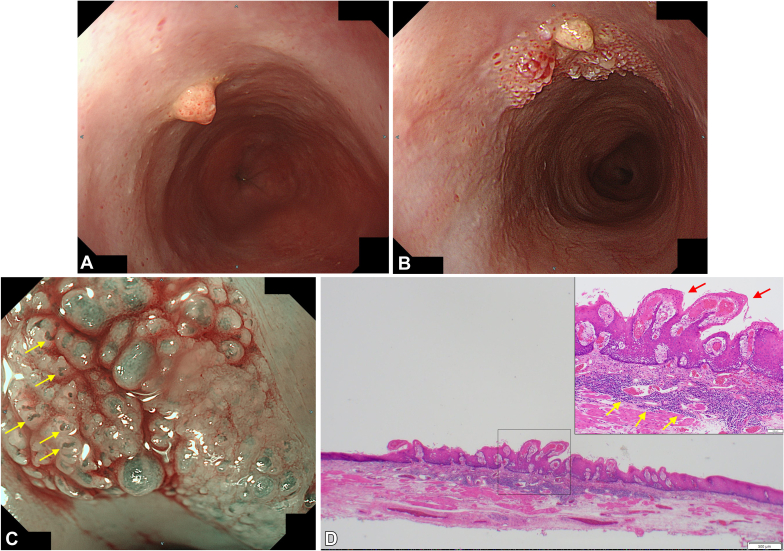
**A,** Initial endoscopy showing a 10-mm, white-colored, elevated lesion on the anal side of the cervical esophageal squamous cell carcinoma. **B,** At 5-month follow-up after chemoradiotherapy, the lesion had markedly enlarged and exhibited a villous morphology. **C,** Magnified endoscopy with narrow-band imaging demonstrated increased caliber and density of intrapapillary capillary loops (*yellow arrows*). **D,** Hematoxylin and eosin (H&E) staining revealed papillary proliferation of stratified squamous epithelium with dilated stromal vessels (*red arrows*) and inflammatory cell infiltration (*yellow arrows*), with no cytological atypia (H&E staining, original magnification ×1; *boxed area*, H&E staining, original magnification ×20).

## Ethical Statement

Ethical approval was not required for this case report.

## Patient consent

The patient in this article has given written informed consent to publication of their case details.

## Disclosure

The authors disclosed no financial relationships.

